# How *Listeria monocytogenes* organizes its surface for virulence

**DOI:** 10.3389/fcimb.2014.00048

**Published:** 2014-04-29

**Authors:** Filipe Carvalho, Sandra Sousa, Didier Cabanes

**Affiliations:** Group of Molecular Microbiology, Unit of Infection and Immunity, Instituto de Biologia Molecular e Celular, University of PortoPorto, Portugal

**Keywords:** *Listeria*, surface proteins, virulence, cell envelope, secretion, protein anchoring domains, regulation

## Abstract

*Listeria monocytogenes* is a Gram-positive pathogen responsible for the manifestation of human listeriosis, an opportunistic foodborne disease with an associated high mortality rate. The key to the pathogenesis of listeriosis is the capacity of this bacterium to trigger its internalization by non-phagocytic cells and to survive and even replicate within phagocytes. The arsenal of virulence proteins deployed by *L. monocytogenes* to successfully promote the invasion and infection of host cells has been progressively unveiled over the past decades. A large majority of them is located at the cell envelope, which provides an interface for the establishment of close interactions between these bacterial factors and their host targets. Along the multistep pathways carrying these virulence proteins from the inner side of the cytoplasmic membrane to their cell envelope destination, a multiplicity of auxiliary proteins must act on the immature polypeptides to ensure that they not only maturate into fully functional effectors but also are placed or guided to their correct position in the bacterial surface. As the major scaffold for surface proteins, the cell wall and its metabolism are critical elements in listerial virulence. Conversely, the crucial physical support and protection provided by this structure make it an ideal target for the host immune system. Therefore, mechanisms involving fine modifications of cell envelope components are activated by *L. monocytogenes* to render it less recognizable by the innate immunity sensors or more resistant to the activity of antimicrobial effectors. This review provides a state-of-the-art compilation of the mechanisms used by *L. monocytogenes* to organize its surface for virulence, with special focus on those proteins that work “behind the frontline”, either supporting virulence effectors or ensuring the survival of the bacterium within its host.

## Introduction

*Listeria monocytogenes* is a ubiquitous Gram-positive bacillus and the causative agent of human listeriosis, a rare foodborne infectious disease with a high and particularly severe incidence in immunocompromised individuals and other risk groups, such as pregnant women and neonates. In these hosts, the invasive form of the illness can be symptomatically manifested as septicemia and meningoencephalitis, or abortions and neonatal infections, which contribute to an estimated mortality rate of 20–30% of clinical cases (Swaminathan and Gerner-Smidt, 2007; Allerberger and Wagner, 2010). The success of this facultative intracellular pathogen results from the ability to promote its own internalization by non-phagocytic cells, which enables the bacterium to overcome important pathophysiological barriers, such as the intestinal epithelium, the blood-brain barrier and the placenta (Lecuit, 2007), and to survive and proliferate inside the host immune phagocytic cells. Decades of studies have contributed to the characterization and comprehension of the *L. monocytogenes* intracellular life cycle (Pizarro-Cerdá et al., 2012). Once internalized, *L. monocytogenes* quickly induces the lysis of its containing vacuole to reach the nutrient-rich cytoplasmic compartment where it can multiply (Gaillard et al., 1987). An actin-based motility machinery allows the bacterium to move in the cytosol and spread to neighboring cells (Ireton, 2013), thus disseminating the infection without re-exposure to the host extracellular immune surveillance.

To efficiently infect cells, *L. monocytogenes* makes use of a large array of virulence effectors that act in one or more steps of the cellular infection cycle (Camejo et al., 2011). The majority of these factors comprise proteins located at the surface of the bacterial cell, in association with the cell envelope or secreted to the extracellular milieu. Their extracytoplasmic localization allows these proteins to interact directly with host cell targets and induce the effects necessary for the establishment of infection. Annotation of the first sequenced genome of *L. monocytogenes* (EGD-e, serotype 1/2a) (Glaser et al., 2001) revealed the presence of 133 genes coding for surface proteins, corresponding to nearly 5% of the complete genome. Interestingly, a comparison with the genome of the phylogenetically close but non-pathogenic *L. innocua* signaled surface proteins as the major difference between both species, highlighting their potential role in *Listeria* pathogenesis (Cabanes et al., 2002). The characterization of this important subset of proteins has allowed us to better understand the role of immediate key virulence effectors of *L. monocytogenes* and to acknowledge the paramount importance of numerous other individual and multicomponent systems of proteins in the promotion and support of their activity.

This review focuses on the various backstage surface players that have been shown to enable *L. monocytogenes* to be fully equipped and proficient as a human pathogen. These include major participants in the mechanisms of surface protein processing and localization, the latter of which also relies on the presence of diverse surface-binding protein motifs or domains. Cell envelope modifications that optimize the surface display of virulence proteins and protect the bacterium from external aggression will be covered, as well as membrane-associated transport systems required for bacterial survival and growth within the host. Finally, relevant content regarding the genetic and post-translational regulation of these surface events will be also addressed.

## Secretion systems

As bacterial surface proteins are being synthesized in the cytoplasm, their surface export signal directs them to the plasma membrane, where a specialized secretion system will assist in their transposition to the other side of the lipid bilayer. Once outside, the protein can then be associated with a cell envelope component, depending on other signals and features encoded in its sequence. Apart from the canonical Sec-dependent pathway, which mediates the secretion of most typical surface proteins in Gram-positive bacteria (Schneewind and Missiakas, 2013), other non-classical secretion systems identified in *L. monocytogenes* include the Tat system, the fimbrilin protein exporter (FPE) system, the flagellar export apparatus (FEA), the Esx-1/Wss system and prophage holins (Desvaux and Hébraud, 2006). So far, only components of the Sec and FEA systems were shown to be required for *Listeria* virulence, although it is believable that further characterization of the other systems will also reveal some degree of contribution to the infectious process.

### Sec system

**Figure 1:**
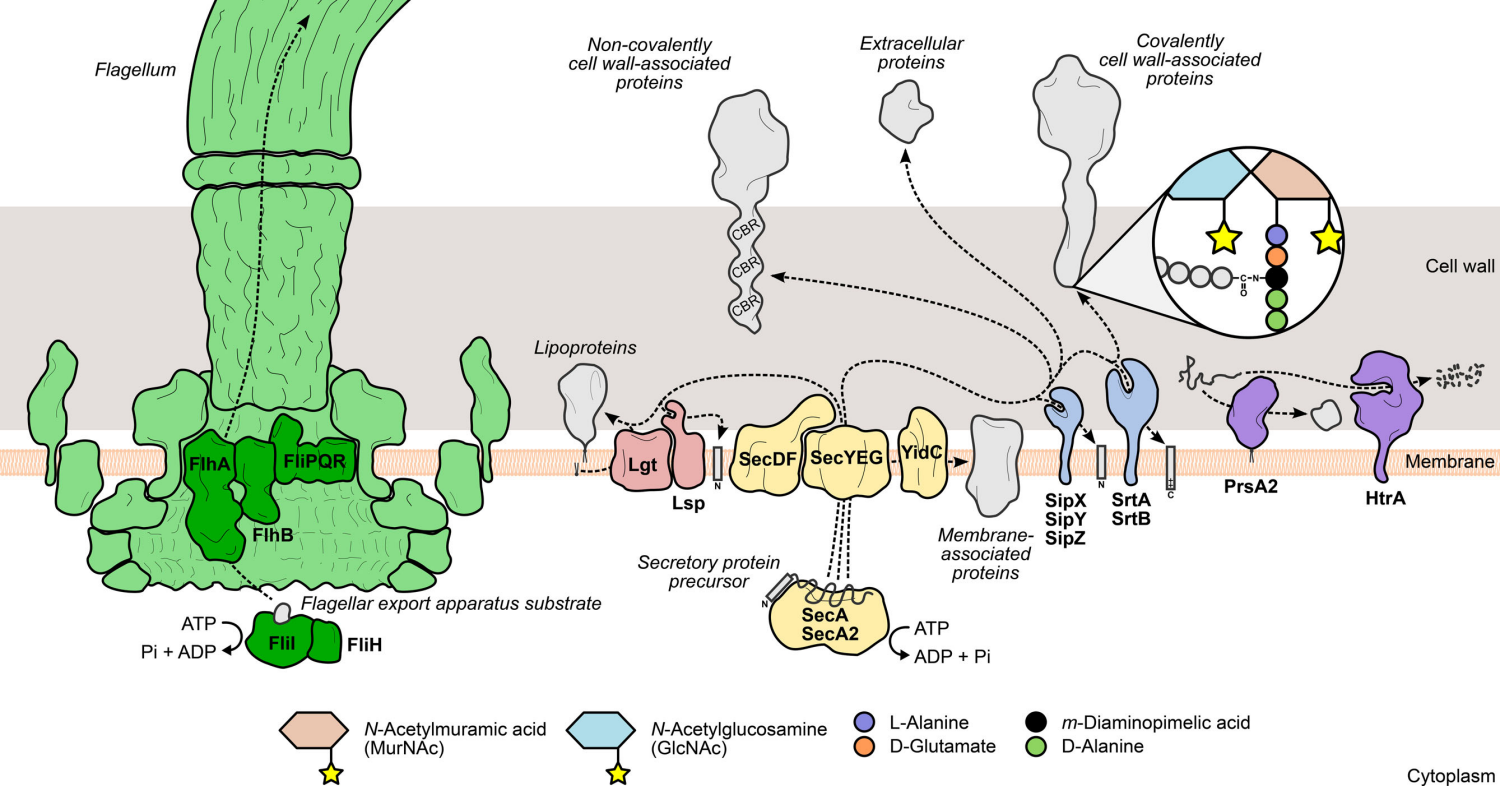
**Mechanisms leading to the surface display of proteins involved in *L. monocytogenes* virulence.** Secretory proteins are exported through the bacterial membrane by the action of specialized secretion systems. The classical Sec system (yellow) recognizes and translocates protein precursors containing N-terminal export signal sequences, which are cleaved afterwards by signal peptidases (Lsp or SipXYZ). Depending on the presence and the nature of surface anchoring domains, the processed protein can become membrane-associated through an anchor molecule (lipoproteins) or by one (hydrophobic tail proteins) or more transmembrane domains (integral membrane proteins). Alternatively, the protein can associate with cell wall components through covalent or non-covalent interactions. Covalently cell wall-anchored proteins require processing of a C-terminal sorting domain by sortases (SrtAB), which cleave an internal signature sequence and append the C-terminus to the stem peptide of peptidoglycan precursors. In contrast, non-covalently anchored proteins associate less stringently with other cell wall components via cell wall-binding repeat-rich domains (CBR). The flagellum (green) assembly machinery has its own export system (dark green), which enables the delivery of rod and filament components to the tip of the nascent flagellar structure. The coordinated activity of surface chaperones and proteases (purple), such as PrsA2 and HtrA, ensures the integrity of the *L. monocytogenes* surfaceome under stressful conditions.

The Sec system is the classical and most important protein translocation system in prokaryotes, enabling the transport of N-terminal signal peptide-containing polypeptides (preproteins) across the cytoplasmic membrane, to be either associated with the cell surface or further released into the extracellular environment. This multimeric system has been thoroughly characterized in *E. coli* and *B. subtilis*, where it comprises a translocon complex of the integral membrane proteins SecYEG, which forms a protein-specific transport channel in the plasma membrane; the peripheral ATP-dependent motor protein SecA, which primes and drives the passage of unfolded substrates through the translocon; and a number of accessory components whose functions include recognition, folding, and membrane integration of translocated proteins (Papanikou et al., 2007; Du Plessis et al., 2011; Chatzi et al., 2013) (Figure 1).

The biological significance of this system is reflected by its striking conservation degree among bacterial species, including *Listeria* spp. (Desvaux and Hébraud, 2006). The requirement of Sec-mediated translocation in the composition of the cell wall and secretory proteomes of *L. monocytogenes* was mostly inferred from bioinformatic predictions coupled with proteomic analyses (Glaser et al., 2001; Calvo et al., 2005; Trost et al., 2005; Bierne and Cossart, 2007). Although informative, these studies did not directly address the operability of the system and the specific contribution of each component to the secretion process. Recently, Burg-Golani et al. tackled this issue by investigating the particular role of the listerial SecDF component, which in *E. coli* mediates the later steps of translocation by assisting the unfolded polypeptide to exit from the translocon channel (Tsukazaki et al., 2011). They identified this membrane protein as a chaperone essential for the secretion and optimal activity of LLO, PlcA, PlcB, and ActA. Accordingly, deletion of SecDF induced defects in phagosomal evasion and cytosolic growth in macrophages, as well as reduced virulence in mice (Burg-Golani et al., 2013).

Unlike Gram-negative species, several Gram-positive bacteria express an additional copy of the SecA ATPase, called SecA2. This paralogous protein fulfills the same role as SecA but, in contrast, is not essential for bacterial viability and possesses a much more limited set of substrates (Rigel and Braunstein, 2008; Bensing et al., 2013). Comparative secretomics implicated *L. monocytogenes* SecA2 in the export of a number of surface and secretory proteins that include known virulence factors, such as the autolysins p60 (or CwhA, previously Iap) and MurA (or NamA) (Lenz and Portnoy, 2002; Lenz et al., 2003) (see Peptidoglycan Turnover), and the fibronectin-binding protein FbpA (Dramsi et al., 2004) (see Unknown Mechanism of Association). Stressing the importance of SecA2-driven secretion in *Listeria* pathogenesis, a Δ*secA2* strain revealed impairment in intercellular spread and reduced virulence in the mouse model. This phenotype partly overlaps with that of a p60 mutant, suggesting that abnormal secretion of this autolysin is a contributing factor to the Δ*secA2* virulence impairment (Lenz et al., 2003). Secretion of OppA, an oligopeptide-binding lipoprotein necessary for optimal replication inside macrophages and in mice organs (Borezee et al., 2000) (see Transport Systems), was shown to be also reduced in the absence of SecA2 (Lenz et al., 2003), although contradicted by more recent data (Renier et al., 2013).

Interestingly, proteins normally found in the cytoplasm (lacking an N-terminal signal peptide) were also identified as SecA2 substrates (Lenz et al., 2003), namely the manganese-dependent superoxide dismutase Sod, which provides bacterial resistance against host-generated toxic oxygen species (Archambaud et al., 2006), and LAP, an alcohol acetaldehyde dehydrogenase required for adhesion to enterocytes under anaerobic conditions (Burkholder et al., 2009). Rather than a direct SecA2-guided export process, the presence of leaderless proteins at the *Listeria* surface could be explained by their passive diffusion through the cell envelope as a result of bacteriolytic events promoted by the signal peptide-containing SecA2 substrates p60 and MurA. In the case of p60, this hypothesis was ruled out since a mutant deficient for this autolysin was found to secrete similar levels of these cytoplasmic proteins as its parental wild type strain (Lenz et al., 2003).

### Flagellar export apparatus

*L. monocytogenes* actively moves in its environment by expressing flagella uniformly around its surface. This motility is temperature-dependent, exhibiting a peak between 20 and 25°C that decreases steadily to a near complete absence of flagella-driven movement at 37°C (Peel et al., 1988). The bacterial flagellum is a highly complex and conserved structure, well-characterized in Gram-negative enteric species. It comprises five main components: (i) the basal body, (ii) the rotor/switch, (iii) the hook and hook/filament junctions, (iv) the filament with its cap, and (v) the FEA (Macnab, 2003, 2004) (Figure 1). The FEA is homologous to a type III secretion system, and once assembled into the membrane core structure, it mediates the translocation of all the external components of the flagellum. The mechanisms through which substrates are recognized by and recruited to the FEA have not yet been elucidated (Macnab, 2003, 2004). The listerial FEA is predicted to transport twelve proteins that make up the hook, rod, and filament structures. The majority of the FEA components characterized in Gram-negative species are encoded in a large flagella/chemotaxis-dedicated gene cluster in *L. monocytogenes* (Desvaux and Hébraud, 2006). Evidence of the contribution of the FEA system toward *L. monocytogenes* virulence was provided by a study on the role of FliI in flagellar biogenesis (Bigot et al., 2005). FliI is a cytosolic ATPase that, in a complex with FliH, binds and carries substrates to the entrance of the export channel, releasing them after ATP hydrolysis (Fan and Macnab, 1996; Minamino et al., 2011). Depletion of FliI in *L. monocytogenes* was shown to abolish flagellar assembly with concomitant loss of bacterial motility. The absence of flagella translated into a dramatic decrease of the levels of adhesion and internalization by epithelial and macrophage cells, albeit with no significant impact on bacterial proliferation inside cells or mouse organs (Bigot et al., 2005).

## Surface protein maturation: processing of precursor polypeptides

Surface proteins exported *via* the Sec system are synthesized as immature polypeptide precursors that undergo post-translational modifications necessary to reach their final location in the cell envelope, where they can fully exert their activity. Preprotein processing generally involves the post-translocational cleavage of signal sequences by specialized surface proteases: N-terminal secretion signal peptides are removed by signal peptidases, while sortases further cleave the C-terminal sorting domain of proteins targeted for covalent binding to the cell wall (Schneewind and Missiakas, 2013). Another type of preprotein modification occurs specifically in lipoproteins and consists in the addition of a lipid anchor to the N-terminal end of the lipoprotein precursor to enable its membrane anchoring (Figure 1). In *L. monocytogenes*, interference with these events results in drastic changes in the surface proteome with negative consequences for bacterial physiology and virulence.

### Signal peptidases

Before completing translocation *via* the Sec system, bacterial proteins are committed to one of two fates: (i) remain integrated within the plasma membrane or (ii) be released to either interact with cell envelope structures or diffuse to the extracellular milieu. The latter situation requires the enzymatic cleavage of the N-terminal signal peptide by a specific family of membrane-bound serine proteases, called signal peptidases (SPases) (Paetzel et al., 2000; Auclair et al., 2012). Depending on features present in their substrates, SPases are categorized into three classes: type I enzymes process canonical unmodified preproteins, while type II members act upon lipid-modified polypeptides (lipoproteins); finally, prepilins are processed by type III SPases (Paetzel et al., 2000).

In contrast with Gram-negative bacteria, which commonly express a single type I SPase, Gram-positive species often encode multiple members of this class (Van Roosmalen et al., 2004). Bioinformatic analysis of the *L. monocytogenes* genome led to the identification of three neighboring genes encoding paralogous type I SPases (SipX, SipY, SipZ) (Bonnemain et al., 2004). Up-regulation of these genes was detected in bacteria isolated from cells infected for 30 min, suggesting a role for these SPases in the early stages of cellular infection (invasion and/or phagosomal escape) (Raynaud and Charbit, 2005). Characterization of strains lacking one or more of these SPases demonstrated that SipZ was essential and sufficient to promote normal levels of protein secretion, bacterial multiplication (in broth and inside cultured cells) and virulence. Particularly, Δ*sipZ* mutants exhibited a significantly reduced secretion of LLO and PC-PLC and a consequent lower hemolytic activity. SipX was also shown to be important for proliferation in mice organs (Bonnemain et al., 2004).

Following membrane translocation, lipoproteins are specifically cleaved by type II SPases that remove the N-terminal signal peptide upstream of a conserved lipid-modified cysteine residue (Nakayama et al., 2012). In *L. monocytogenes*, Lsp, a lipoprotein-specific SPase, was identified and deletion of its encoding gene gave rise to bacteria deficient in lipoprotein processing, which ultimately impaired their capacity to escape the phagosomal compartment and significantly attenuated their virulence. Immature and alternatively processed forms of the lipoprotein LpeA were detected in Δ*lsp* bacteria (Réglier-Poupet et al., 2003a). Considering that LpeA was shown to promote *L. monocytogenes* entry into non-phagocytic cells, these presumably non-functional LpeA variants might partly contribute for the reduction in host cell invasion associated with this mutant strain (Réglier-Poupet et al., 2003b) (see Lipobox Motif).

### Lipoprotein lipidation

The Lsp-mediated processing of an N-terminal signal peptide-containing lipoprotein typically occurs after the newly synthesized polypeptide has been modified with a membrane-anchoring lipid. This step is catalyzed by an enzyme called Lgt (for lipoprotein diacylglycerol transferase), which catalyzes the covalent linkage of a phospholipid-derived diacylglycerol moiety to the sulfhydryl group of a cysteine residue located in a lipobox motif, at the end of the signal peptide (Kovacs-Simon et al., 2011). Whereas Lgt activity is crucial for the growth of Gram-negative bacteria, the same does not necessarily occur in certain Gram-positive genera, such as Firmicutes (Hutchings et al., 2009). Indeed, *L. monocytogenes* lipoproteins could still have their signal peptide removed by Lsp without previous Lgt-mediated lipidation, thus suggesting a less strict pathway for lipoprotein maturation (Baumgärtner et al., 2007). Regarding its role in *Listeria* virulence, Lgt was described as being specifically required for replication inside eukaryotic cells, due to its role in the maturation of several proteins involved in the transport of substrates from the host cell cytosol. In fact, comparative secretomic studies revealed over 20 different lipoproteins that were solely or increasingly secreted in the absence of Lgt, the large majority of which comprised putative ABC transporter components associated with intake of nutrients (see Nutrient Uptake) and substrate sensing systems (Baumgärtner et al., 2007).

### Sortases

To become stably associated with the Gram-positive cell wall, surface protein precursors have to be processed by an enzyme called sortase A. This trans-cytoplasmic, membrane-bound transpeptidase recognizes polypeptides with a C-terminal sorting signal sequence containing a signature LPXTG motif (Schneewind et al., 1992). Sortase A then promotes cell wall anchoring of these proteins via a two-step mechanism: (i) the catalytic site cysteine breaks the peptide bond between the threonine and glycine residues of the LPXTG motif, forming an intermediate protein-sortase complex linked by a thioester bond; (ii) the amine group of a muropeptide (peptidoglycan precursor) attacks the thioester bond, releasing the cleaved protein from the sortase A active site, and forms a new amide bond with the carboxyl group of the new C-terminal threonine (Ton-That et al., 1999) (Figure 1). The ubiquity of LPXTG proteins and the conservation of the sorting motif were investigated and validated across a wide range of Gram-positive species (Navarre and Schneewind, 1999; Pallen et al., 2001; Comfort and Clubb, 2004), highlighting the importance of this sortase A-mediated anchoring mechanism in Gram-positive physiology. The virulence of Gram-positive pathogens is also dependent of sortase activity, as many virulence factors are expressed as cell wall-anchored surface proteins (Navarre and Schneewind, 1999). Bioinformatics analysis of the sequenced *L. monocytogenes* genome predicted the existence of a large number of putative LPXTG protein-encoding genes (Cabanes et al., 2002). Soon after, the listerial homolog of sortase A was identified and shown to be of chief importance for the surface anchoring of internalin A (InlA), one of the two major invasion-promoting proteins, and consequently for bacterial entry into eukaryotic cells and full virulence in mice (Bierne et al., 2002; Garandeau et al., 2002). In the following years, the role of sortase A in *L. monocytogenes* infection became more important as several novel virulence effectors were found to be associated with the bacterial surface through the activity of this enzyme (see LPXTG and NXXTX Sorting Signals).

A second sortase-encoding gene, *srtB*, was identified in *L. monocytogenes* in a locus far apart from *srtA*. It encodes a protein 23% identical with sortase A and with sortase-like motifs (Bierne et al., 2002, 2004). The existence of two or more sortase paralogs is not uncommon in Gram-positive bacteria (Pallen et al., 2001), and, in particular, sortase B orthologs are expressed in other species, such as *S. aureus* and *B. anthracis* (Zhang et al., 2004). Similarly to *S. aureus* (Mazmanian et al., 2001), the two listerial sortases do not display overlapping or redundant activities, indicating that they act upon different classes of substrates. Moreover, the substrate spectrum of sortase B is more limited than that of sortase A, with only two proteins identified as sortase B substrates (Bierne et al., 2004; Pucciarelli et al., 2005) (see LPXTG and NXXTX Sorting Signals). Sequence alignment of several known and putative sortase B substrates indicated that the enzyme recognizes an NXXTX consensus motif sequence. Whereas *L. monocytogenes* Δ*srtA* mutants are significantly less virulent, the inactivation of *srtB* yields no effect, indicating that sortase B-processed proteins have no role in listerial virulence (Bierne et al., 2004).

## Surface protein localization: anchoring domains

Once surface proteins have been translocated, they are able to associate with components of the cell envelope *via* specific binding domains encoded in their sequence. For instance, sortase substrates contain a characteristic C-terminal sorting domain that allows covalent attachment to the peptidoglycan. On the other hand, proteins containing domains with tandem repeated sequences often display a more labile interaction with secondary cell wall components (e.g., glycopolymers). In addition, proteins may also be tethered to the plasma membrane by a lipid anchor or completely span the bilayer, provided that they contain adequate stretches of hydrophobic residues to act as transmembrane regions (Figure 1). Notwithstanding, proteins lacking recognizable surface-binding sequences have been found associated with the bacterial surface through mechanisms that still require elucidation.

### Cell wall association

#### LPXTG and NXXTX sorting signals

The precursors of proteins covalently anchored to the Gram-positive cell wall by a sortase A-dependent mechanism feature a C-terminal sorting signal of about 30–40 residues comprising (i) an LPXTG pentapeptide motif, (ii) a hydrophobic domain, and (iii) a short positively charged tail (Schneewind et al., 1992). Whereas the hydrophobic and charged domains of the sorting signal can display variability in their sequence and/or length, the LPXTG motif is very conserved (Fischetti et al., 1990; Schneewind et al., 1992). Studies with C-terminal truncates of staphylococcal protein A revealed that proper cell wall anchoring requires a complete sorting signal, and hinted that the hydrophobic and charged residues downstream of the LPXTG motif are responsible for retaining the polypeptide in the bacterial membrane until its recognition by sortase A (Schneewind et al., 1992, 1993). The LPXTG motif is accommodated in the sortase A active site, where a catalytic cysteine initiates cleavage of the peptide bond between the threonine and the glycine residues. The cleaved protein becomes temporarily bound to the sortase (Ton-That et al., 1999), which seems to prevent its diffusion to the extracellular medium. The protein is then transferred to its final acceptor, lipid II (peptidoglycan precursor), which establishes a new bond between the amine group of a cross-bridge residue (*meso*-diaminopimelic acid in *L. monocytogenes*) and the C-terminal threonine carboxyl group of the surface protein (Ton-That et al., 1997). Proteins with LPXTG motifs are found in a multiplicity of Gram-positive organisms (Navarre and Schneewind, 1999; Mazmanian et al., 2001; Hendrickx et al., 2009; Pérez-Dorado et al., 2012). *L. monocytogenes* stands out as the species with the largest number, encoding 41 proteins (over 1% of its genome) (Glaser et al., 2001; Cabanes et al., 2002), seven of which are currently described as virulence factors. InlA, important for entry into epithelial cells and virulence in mice (Gaillard et al., 1991; Lingnau et al., 1995), was the first to be identified, long before the *L. monocytogenes* genome was sequenced. The list comprises four other internalin family members (Bierne and Cossart, 2007)—InlF (Kirchner and Higgins, 2008), InlH (Pucciarelli et al., 2005; Personnic et al., 2010), InlJ (Sabet et al., 2005, 2008), and InlK (Dortet et al., 2011)—with roles in host cell adhesion and immune evasion, and two non-internalins, Vip (Cabanes et al., 2005) and LapB (Reis et al., 2010), important for entry into cells.

A subset of covalently attached cell wall proteins feature a different sorting motif, characterized by an NXXTX consensus sequence that targets surface protein precursors for sortase B processing (Comfort and Clubb, 2004; Mariscotti et al., 2009). Sortase B enzymes have few substrates, which are usually encoded by genes arranged in an operon together with *srtB* (Marraffini et al., 2006). Interestingly, they are involved in heme-iron scavenging and uptake (Mazmanian et al., 2002; Maresso and Schneewind, 2006; Xiao et al., 2011; Klebba et al., 2012), indicating that the sortase B anchoring mechanism may have evolved differently from sortase A to become more specialized in the anchoring of proteins required for iron homeostasis. *L. monocytogenes* encodes only two proteins with NXXTX motifs (Bierne et al., 2004), both of which require sortase B for cell wall anchoring (Pucciarelli et al., 2005). One of them, SvpA, is a surface-associated protein required for iron uptake and bacterial persistence in mouse organs (Newton et al., 2005). The other listerial sortase B substrate, Lmo2186, possesses two putative sorting motifs, NKVTN and NPKSS (underlined residue is common to both), but only the latter is necessary for surface anchoring (Mariscotti et al., 2009). SvpA was first characterized as a virulence factor, as its depletion resulted in deficient escape from macrophage phagosomes (Borezée et al., 2001). However, more recent data indicate that neither SvpA nor Lmo2186 are used by *L. monocytogenes* to promote infection (Newton et al., 2005), agreeing with results demonstrating that sortase B is dispensable for virulence (Bierne et al., 2004).

#### GW module

Many surface proteins interact non-covalently with the cell wall through a domain containing a variable number of tandemly arranged sequences, called GW modules, whose name derives from the presence of a conserved glycine (G)-tryptophan (W) dipeptide. This cell wall association motif was first discovered in *L. monocytogenes* InlB (Braun et al., 1997), an internalin-like protein that promotes entry into hepatocytes, epithelial, and endothelial cells (Dramsi et al., 1995; Lingnau et al., 1995; Parida et al., 1998). InlB contains three GW modules in its C-terminal cell wall association domain (GWA), which are required and sufficient to confer cell wall-binding properties to the protein (Braun et al., 1997). InlB variants lacking the GWA are unable to associate to the surface of non-invasive *Listeria* and promote their entry into eukaryotic cells (Braun et al., 1998). Structural analysis of the GW module revealed an interesting resemblance with SH3 domains, known to be involved in protein-protein interaction in signal transduction pathways, but steric hindrance discarded a functional SH3-like activity for GW modules (Marino et al., 2002). Lipoteichoic acids (LTAs) were identified as the “surface anchor” of InlB, binding to its GWA. The interaction with these cell envelope glycopolymers is highly specific, as LTAs from *L. innocua* or *S. pneumoniae* are not able to capture InlB (Jonquières et al., 1999). The GWA of InlB also enables its association with glycosaminoglycans present at the surface of host cells and with the receptor of the complement C1q globular part (gC1q-R), significantly potentiating InlB-mediated invasion (Braun et al., 2000; Jonquières et al., 2001; Banerjee et al., 2004; Asano et al., 2012). The binding strength of proteins containing GW modules is proportional to the number of modules. This is illustrated by comparing the surface association levels of InlB and Ami, another GW protein with autolytic activity and an important role in bacterial adhesion to host cells (Milohanic et al., 2000, 2001; Asano et al., 2012). Containing eight GW modules, Ami is found exclusively in association with the bacterial surface, whereas InlB (only three modules) is detected in the cell envelope and secreted fractions (Braun et al., 1997). *L. monocytogenes* encodes seven other GW proteins, all of which have a predicted amidase domain in common with Ami (Cabanes et al., 2002), hinting that they also may possess autolytic functions. Indeed, one of them, Auto, was described to behave also as an autolysin (Cabanes et al., 2004) (see Peptidoglycan Turnover). Staphylococcal autolysins are also associated to the bacterial surface via structural motifs resembling the listerial GW modules (Oshida et al., 1995; Heilmann et al., 1997; Hell et al., 1998; Allignet et al., 2001), strongly suggesting that this cell wall association protein motif has evolved with the specific purpose of mediating the reversible surface binding of proteins with autolytic activity (Milohanic et al., 2001).

#### LysM domain

Lysin motif (LysM) domains are encountered in proteins from a broad variety of organisms, such as plants, fungi, bacteria, and viruses (Buist et al., 2008). Initially found in bacterial and phage lysins, from which the motif took its name (Birkeland, 1994), the LysM domain is characterized by a variable number of roughly 40–80-residue repeats, spaced by stretches rich in serine, threonine, and asparagine (Buist et al., 1995). The consistent presence of this domain in proteins expressing cell wall-degrading activity suggested that LysM repeats are important for retention of these enzymes within the peptidoglycan (Joris et al., 1992; Birkeland, 1994). This hypothesis was validated through binding studies using the LysM domains of *Lactococcus lactis* and *Enterococcus faecalis* autolysins (Steen et al., 2003; Eckert et al., 2006). Further studies singled out *N*-acetylglucosamine (GlcNAc) as the peptidoglycan moiety bound by LysM (Buist et al., 2008). However, instead of an expected uniform surface distribution, many LysM-containing proteins appear localized to specific sites by the excluding action of cell wall components, such as LTAs (Steen et al., 2003), or modifications, such as *O*-acetylation (Veiga et al., 2007) (see Modification of Cell Envelope Components). LysM domains are found in six *L. monocytogenes* proteins (Bierne and Cossart, 2007), two of which, p60 and MurA, have been characterized as autolysins with a relevant role in infection (Lenz et al., 2003) (see Peptidoglycan Turnover). The p60 sequence contains a C-terminal NlpC/p60 domain putatively responsible for the peptidoglycan peptidase activity (Anantharaman and Aravind, 2003; Layec et al., 2008), and an N-terminal region with two LysMs separated by an SH3-like domain (Bierne and Cossart, 2007), which presumably mediate protein binding to peptidoglycan. Unlike p60, MurA contains four C-terminal LysM repeats (Carroll et al., 2003), which may be important to position the catalytic site of this autolysin in a manner distinct of p60, so as to optimize its activity. A third LysM protein of *L. monocytogenes* (Lmo2522) was recently characterized as one of two novel listerial resuscitation-promoting factors, i.e., muralytic enzymes important for jump-starting the growth in dormant bacteria (Pinto et al., 2013).

### Membrane association

#### Lipobox motif

Bacterial lipoproteins contribute to important physiological roles, such as substrate binding and transport, antibiotic resistance, signaling, and folding of secreted proteins (Sutcliffe and Russell, 1995; Hutchings et al., 2009), and were also shown to take an active part in virulence-associated processes, such as adhesion, invasion, and immunomodulation (Kovacs-Simon et al., 2011; Nakayama et al., 2012). As described above, lipoproteins are expressed as immature polypeptides, which are converted to prolipoproteins by the addition of a lipid moiety at a specific motif in the distal portion of the N-terminal signal peptide. This motif, called lipobox, is characterized by a four-residue sequence containing a conserved cysteine (Sutcliffe and Harrington, 2002; Babu et al., 2006). The sulfhydryl group of the cysteine establishes a thioester bond with phospholipid-derived diacylglycerol in a reaction catalyzed by Lgt (Kovacs-Simon et al., 2011). The N-terminal lipid group inserts into the outer leaflet of the lipophilic plasma membrane, thus enabling the retention of the protein at the cell surface once the signal peptide is cleaved. In *L. monocytogenes*, the biological importance of lipoproteins is emphasized by their preponderance in the surface proteome: 68 of 133 surface proteins were predicted to be lipoproteins, based on the presence of an N-terminal lipobox (Glaser et al., 2001), and 26 were later confirmed experimentally (Baumgärtner et al., 2007). Interestingly, nearly half of the listerial lipoproteins are presumed to act as substrate-binding components of ABC transporter systems (Bierne and Cossart, 2007), performing the equivalent functions of periplasmic solute-binding proteins in Gram-negative bacteria (Tam and Saier, 1993). Such is the case of the above-mentioned lipoproteins OppA, which participates in the oligopeptide uptake, and LpeA, which belongs to the LraI family of manganese-importing ABC transporter components (Novak et al., 1998), although supporting evidence for this function in *L. monocytogenes* have yet to be obtained. Another substrate-carrying lipoprotein, OpuC, operates in the transport of L-carnitine, important for bacterial osmotolerance and, without which, *L. monocytogenes* is unable to efficiently persist in mice organs (Sleator et al., 2001) (see Osmolyte Uptake and Bile Acid Extrusion). Fifteen other lipid-anchored proteins were predicted to have enzymatic activities (Bierne and Cossart, 2007). Among them, the best studied and with a significant contribution to infection is the surface chaperone PrsA2 (see Chaperones).

#### Hydrophobic tail

Surface proteins can also be associated to the bacterial membrane through an N- or C-terminal tail comprised of hydrophobic residues that spans and stably inserts the protein in the lipid bilayer, during translocation. The orientation of the proteins in the membrane is pre-determined by the presence and localization of positively charged residues relative to the membrane-spanning domain (stop-transfer signals) (Dalbey et al., 2011). From the ten predicted *L. monocytogenes* surface proteins with a putative C-terminal hydrophobic tail (Bierne and Cossart, 2007), only ActA has been biochemical and functionally characterized (Domann et al., 1992; Kocks et al., 1992). This key virulence factor promotes intracellular motility and intercellular spread by triggering the polymerization of host cell actin into a comet-like tail of actin filaments that propels the bacterium through the cytoplasm, toward neighboring cells (Kocks et al., 1995; Monack and Theriot, 2001). ActA was also shown to enable *L. monocytogenes* to escape autophagy (Yoshikawa et al., 2009) and to play a key role in persistence within the host and transmission from the host back to the environment (Travier et al., 2013). A large number of listerial enzymes linked with cell wall metabolism and surface protein processing—such as sortases (Mazmanian et al., 2000), signal peptidases (Paetzel et al., 2000) and penicillin-binding proteins (PBPs, see Peptidoglycan Assembly)—are anchored to the bacterial membrane by an N-terminal hydrophobic tail (Bierne and Cossart, 2007), which in many cases corresponds to a signal peptide sequence lacking a type I cleavage site.

### Unknown mechanism of association

Several proteins secreted by *L. monocytogenes* lack recognizable surface-targeting sequences and a number of them are associated with the cell envelope despite having no predicted surface-binding domains (Schaumburg et al., 2004; Trost et al., 2005). Consistent and, in some cases, significant secretion of the same proteins in different studies seems to discard or at least minimize the contribution of bacterial cell lysis to their extracytoplasmic localization. In turn, it suggests that they use a non-classical type of secretion mechanism (Schaumburg et al., 2004). So far, the only example of an unconventionally secreted and surface-associated protein with a described virulence-promoting function in *L. monocytogenes* is FbpA. Like many streptococcal fibronectin-binding proteins, FbpA lacks all the classical cell surface sorting and anchoring sequences, yet it was detected in the bacterial plasma membrane after subcellular fractionation. It was shown to facilitate *in vitro* adhesion to hepatocytes and to support liver infection in mice (Dramsi et al., 2004).

## Surface protein quality control

The accumulation of non-natively configured or damaged proteins poses a threat to cell viability as it may lead to the formation of toxic aggregates. To prevent this, bacteria have evolved mechanisms of protein quality control, which rely on the coordinated activity of molecular chaperones and proteases to repair misfolded substrates, degrade irreparably damaged molecules and activate stress response pathways (Wickner et al., 1999).

### Chaperones

Sec-mediated export requires that proteins are kept in an unfolded configuration to pass through the translocase channel (Desvaux and Hébraud, 2006). Immediately after transposing the membrane, proteins must properly fold into their native conformation to acquire their activity and become less susceptible to proteolytic attacks, which occur at a high frequency in the extracytoplasmic environment (Sarvas et al., 2004). A specific group of ATP-dependent chaperones, called foldases, assist in the correct post-translocational folding of secreted proteins. The number and variety of these enzymes is another distinguishing element between Gram-negative and Gram-positive bacteria. Whereas the former are known to express multiple foldases with different selectivities (Merdanovic et al., 2011), the latter species are not as well-supplied (Sarvas et al., 2004). PrsA, a ubiquitous Gram-positive lipoprotein with peptidyl-prolyl *cis*-*trans* isomerase activity (Drouault et al., 2002), was originally identified in *B. subtilis*, where mutations in its encoding gene were responsible for deficient secretion of exoproteins (Kontinen et al., 1991). Critical for *B. subtilis* viability, PrsA was shown to have also chaperone activity, promoting optimal secretion levels without influencing protein translocation, and prevented the degradation of exported proteins (Vitikainen et al., 2001). *L. monocytogenes* encodes two PrsA homologs, PrsA1 and PrsA2, neither of which are essential for bacterial survival. Despite highly similar amino acid sequences, only PrsA2 displayed chaperone activity comparable to the PrsA of *B. subtilis* (Alonzo et al., 2009; Alonzo and Freitag, 2010) (Figure 1). Up-regulation of *prsA2* was detected in *L. monocytogenes* isolated from infected macrophages (Chatterjee et al., 2006), suggesting a role for PrsA2 in the adaptation to the host intracellular environment. This increased expression is not directly controlled by PrfA, the major virulence gene regulator (Zemansky et al., 2009) (see Transcriptional Regulation), in spite of the presence of a putative PrfA-binding sequence upstream of *prsA2* (Glaser et al., 2001) and data showing increased PrsA2 secretion in strains expressing hyperactive forms of PrfA (Port and Freitag, 2007). Further studies confirmed the involvement of PrsA2 in intracellular replication, cell-to-cell spread and virulence in a mouse model (Chatterjee et al., 2006; Alonzo et al., 2009). In particular, PrsA2 is determinant for the proper secretion and activity of major virulence factors, such as listeriolysin O (LLO), metalloprotease (Mpl) and phosphatidylcholine-specific phospholipase C (PC-PLC) (Alonzo et al., 2009; Zemansky et al., 2009; Forster et al., 2011).

### Proteases

The high temperature requirement A (HtrA) family of proteases is one of the most conserved in all living organisms and the most predominant group of bacterial extracytoplasmic proteases (Figure 1). Some HtrA proteases, like the archetypal *E. coli* DegP (Krojer et al., 2008), also exhibit ATP-independent chaperone activity (Clausen et al., 2002), and this dual role is important for other biological processes, such as activation of stress responses and virulence (Clausen et al., 2011). Indeed, HtrA was shown to promote host cell invasion and survival inside macrophage phagosomes in a number of bacterial pathogens, such as *Helicobacter pylori*, *Yersinia enterocolitica*, and *Salmonella enterica* serovar Typhimurium (Ingmer and Brøndsted, 2009; Hoy et al., 2010). A similar intracellular phenotype was also observed in HtrA-depleted *L. monocytogenes* mutants (Stack et al., 2005; Wilson et al., 2006), indicating that a functional HtrA is crucial for bacterial survival in the stress-inducing environment of the macrophage phagosome, possibly by promoting the degradation and preventing the accumulation of stress-damaged proteins. HtrA was also shown to be required for efficient colonization of mouse organs (Stack et al., 2005; Wilson et al., 2006). Interestingly, like PrsA2, up-regulation of *htrA* was detected in intracellular bacteria (Chatterjee et al., 2006) and higher amounts of the protein were secreted by a *L. monocytogenes* strain expressing a constitutively active variant of PrfA (Port and Freitag, 2007).

## Cell envelope metabolism

The cell envelope is a fundamental and defining structure of prokaryotes. In Gram-positive bacteria, it comprises the plasma membrane and a cell wall, which provides physical support and protection against external aggression. Its mesh-like constituent, the peptidoglycan, also acts as a biological scaffold for the surface positioning of proteins and other glycopolymers with relevant physiological roles. The remodeling of the cell wall is vital for bacterial growth and division, and requires a dynamic balance between peptidoglycan assembly and turnover. Coordination between these processes is mandatory to prevent morphological malformations and concomitant functional defects, such as the mislocalization of surface molecules.

From an immunological view, the cell wall is a particularly relevant structure. Cell wall turnover events can generate fragments that are specifically recognized by and activate the host innate immune system. In turn, innate immunity effectors, such as lysozyme and cationic peptides, target the cell wall to promote bacterial lysis. The introduction of specific modifications in components of the cell envelope is a strategy developed by bacteria to render them undetectable to both immune recognition and to the bacteriolytic activity of host defense enzymes and peptides (Davis and Weiser, 2011).

### Peptidoglycan assembly

**Figure 2:**
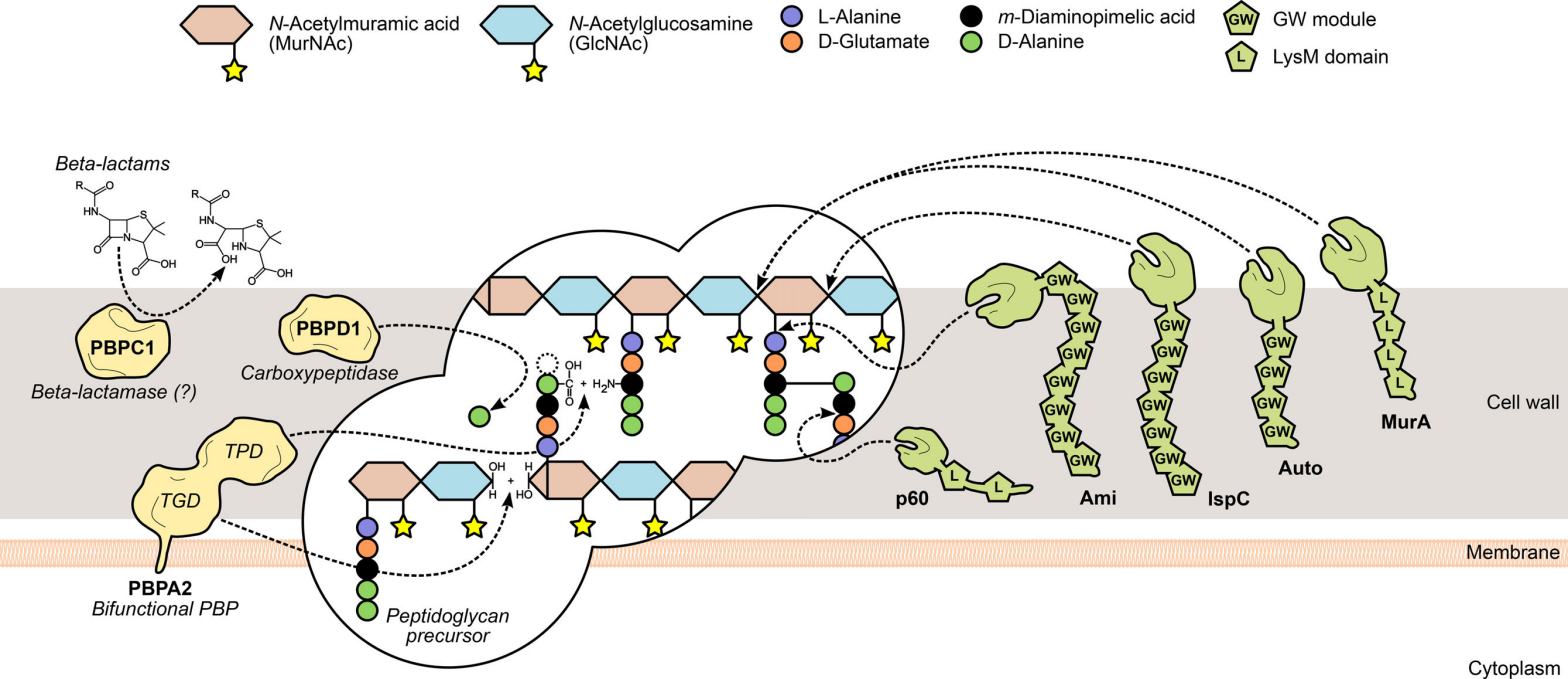
***L. monocytogenes* peptidoglycan metabolism and the surface proteins involved in its assembly and turnover.** The peptidoglycan sacculus is polymerized with cytoplasmic precursors with the help of penicillin-binding proteins (PBPs, yellow). High-molecular-weight PBPs, such as PBPA2, contain transglycosylase (TGD) and transpeptidase domains (TPD) that catalyze, respectively, glycan chain elongation and stem peptide bridging between adjacent chains. Other PBPs include the low-molecular-mass carboxypeptidases, which cleave the terminal D-alanyl-D-alanine stem peptide bond (e.g., PBPD1), and beta-lactamases, which degrade PBP-inhibiting antibiotics to promote bacterial survival (e.g., PBPC1). On the other hand, the degradation of mature peptidoglycan, during bacterial elongation/division or autolysis, is mediated by autolysins (green), a family of surface hydrolases that can cleave the peptidoglycan at different sites: within the glycan chain (*N*-acetylglucosaminidases or *N*-acetylmuramidases) or the stem peptide (endo- and carboxypeptidases), or between both (*N*-acetylmuramoyl-L-alanine amidases). Interestingly, autolysins commonly associate non-covalently with the bacterial surface *via* cell wall-binding repeats, such as the GW modules in Ami, Auto and IspC, or the LysM repeats in MurA and p60.

Peptidoglycan is assembled outside of the bacterial cell through the polymerization and bridging of subunits generated on the cytoplasmic side of the membrane. Following translocation, these building blocks are transferred and integrated into existing peptidoglycan chains by the action of a multifunctional family of surface proteins called PBPs (Figure 2). These membrane-anchored proteins are categorized into high molecular weight (HMW) PBPs—the major players in peptidoglycan assembly—and low molecular weight (LMW) PBPs, both of which are characterized by the presence of an archetypal DD-peptidase domain (Macheboeuf et al., 2006). In HMW PBPs, the peptidase domain is located at the C-terminus and catalyzes the crosslinking of adjacent glycan strands through their subunit stem peptides (transpeptidation). Additionally, they may contain an N-terminal domain that displays transglycosylase activity, necessary for the elongation of *N*-acetylglucosamine (GlcNAc)-*N*-acetylmuramic acid (MurNAc) glycan strands. LWM PBPs perform roles linked to peptidoglycan maturation and recycling (Macheboeuf et al., 2006; Sauvage et al., 2008). The peptidase domain cleaves the D-Ala-D-Ala bond of a peptidoglycan subunit stem peptide, releasing the terminal alanine residue and forming a new bond between the remaining alanine and the diamino acid residue from a stem peptide in a different strand. Penicillin and other β-lactam compounds take advantage of their structural similarity with the D-Ala-D-Ala dipeptide to bind irreversibly to and inhibit most PBPs, thus promoting bacterial death by perturbing cell wall synthesis (Tipper and Strominger, 1965; Ghuysen, 1994). *In silico* studies have allowed the identification of ten PBP-like protein-encoding genes in *L. monocytogenes* (Guinane et al., 2006; Korsak et al., 2010) and β-lactam-binding assays confirmed that nine of them expressed functional PBPs (Korsak et al., 2010). They comprise five HMW proteins—class A members PBPA1 and PBPA2 (former PBP1 and PBP4) and class B members PBPB1, PBPB2 (former PBP3 and PBP2) and PBPB3—and four LMW PBPs, including the carboxypeptidase PBPD1 (former PBP5) and two β-lactamases (Korsak et al., 2010). Studies on listerial PBPs have largely focused on their biochemical characterization, namely through the determination of their affinity to several β-lactam derivatives (Gutkind et al., 1990; Pierre et al., 1990; Vicente et al., 1990; Guinane et al., 2006; Zawadzka-Skomial et al., 2006). In some cases, mutational approaches allowed the elucidation of the roles of some PBPs toward *L. monocytogenes* virulence. For instance, PBPB1, PBPD1, but mostly PBPA2 and PBPC1, were found to be important for the colonization of the mouse spleen (Guinane et al., 2006). The depletion of these PBPs resulted in variable degrees of morphological defects (Guinane et al., 2006; Korsak et al., 2010), and the pleiotropic effects elicited by such modifications are likely to be responsible for the attenuated virulence.

### Peptidoglycan turnover

The reshaping of the bacterial cell wall is vital for many physiological processes, particularly cell growth and division, and thus depends on a dynamic equilibrium between the degradation and recycling of cell wall components (Popowska, 2004; Vollmer et al., 2008). Peptidoglycan turnover relies on the activity of another family of surface-associated enzymes, called autolysins, which catalyze the hydrolysis of every existing covalent bond in the mature peptidoglycan matrix. The nature and location of the bond(s) cleaved by an autolysin is determined by its functional specificity within the broader family of peptidoglycan hydrolases (Vollmer et al., 2008). *N*-acetylglucosaminidases (NAGases) and *N*-acetylmuramidases (NAMases) cleave the glycosidic bond between glycan chain residues GlcNAc and MurNAc, respectively, after GlcNAc and MurNAc; *N*-acetylmuramyl-L-alanine amidases (or simply amidases) separate the stem peptide from the sugar strand by breaking the bond between MurNAc and L-alanine; finally, endo- and carboxypeptidases hydrolyse the amide bonds within and between stem peptides (Vollmer et al., 2008) (Figure 2). The existence of multiple autolysins sharing the same activity and substrate attests for the functional redundancy associated with peptidoglycan hydrolases, which has complicated the characterization of the role of individual cell wall-degrading enzymes.

The genome of *L. monocytogenes* EGD-e is predicted to encode six NAGases, four NAMases, four amidases, and a multiplicity of peptidoglycan peptidases, but only a few have been experimentally validated (Popowska, 2004; Bierne and Cossart, 2007; Pinto et al., 2013). The only predicted NAGases with confirmed peptidoglycan hydrolase activity are MurA and Auto, although their specific NAGase activity remains to be verified (Carroll et al., 2003; Cabanes et al., 2004). MurA is necessary for proper cell separation during growth and its absence or malfunction results in virulence defects, namely in adhesion to host cells (Lenz et al., 2003; Alonzo et al., 2011). Auto is important for entry into non-phagocytic cells and virulence in mice and guinea pigs (Cabanes et al., 2004). The contribution of both autolysins toward *Listeria* virulence occurs possibly through different mechanisms. This is suggested by their distinct cell wall association domains—MurA contains LysM repeats, Auto has GW modules—which hint at a differential cell wall localization, and their relative importance for cell wall remodeling, as *murA* mutants cannot separate well and grow in filaments, while *aut* mutants maintain a normal morphology (Carroll et al., 2003; Cabanes et al., 2004). Two putative *L. monocytogenes* amidases contain C-terminal GW module repeats, suggesting similar surface association requirements, and among them is the virulence-promoting adhesin Ami (see GW Module). Although none of the NAMases have been deeply characterized in a virulence-oriented perspective, two were recently shown to possess lysozyme-like activity in the presence of cell wall substrate and to be required for stimulating the replication of quiescent bacteria, possibly through their impact in cell wall reshaping and thus in cell growth and division (Pinto et al., 2013). On the other hand, IspC, a putative NAMase-like protein with a highly significant contribution to *Listeria* infection, was identified in a serotype 4b strain (Wang and Lin, 2007, 2008). Interestingly, IspC mutants were not affected in their growth *in vitro* and cell morphology, but showed cell type-dependent defects in nearly every step of the cellular infection cycle (Wang and Lin, 2008).

Common to many peptidoglycan hydrolases is the presence of an NlpC/p60 domain, related to the CHAP (cysteine, histidine-dependent amidohydrolase/peptidase) superfamily. Interestingly, most NlpC/p60 proteins are found in the genus *Bacillus* and *Listeria*, but not in *Staphylococcaceae*, which express proteins with another CHAP-type domain (Bateman and Rawlings, 2003; Layec et al., 2008). This is most likely a reflection of the affinity of the NlpC/p60 domain for the γ-D-glutamyl-*meso*-diaminopimelic acid bond (Rigden et al., 2003), which is replaced by a γ-D-glutamyl-L-lysine linkage in staphylococci. Four *L. monocytogenes* proteins contain putative NlpC/p60 domains and were predicted to possess cell wall hydrolase activity (Bierne and Cossart, 2007). Two of them, p45 (or Spl) and p60, have been studied and their function validated. Spontaneous mutants that secreted lower amounts of this protein, also known as CwhA (cell wall hydrolase A), showed a filamentous morphology and reduced host cell invasion efficiency, suggesting that p60 was required for entry into non-phagocytic cells (hence its first name, Iap, for invasion-associated protein). Indeed, exogenously added p60 not only restored the invasiveness potential (Kuhn and Goebel, 1989), but also disrupted the bacterial chains into individual cells, due to its cell wall-degrading activity (Wuenscher et al., 1993). Lack of functional p60 results in septum abnormalities that disrupt actin-based intracellular motility, impairing optimal cell-to-cell spread and, overall, virulence (Hess et al., 1996; Pilgrim et al., 2003; Faith et al., 2007). The immunomodulatory properties of p60 have been previously addressed (Pamer, 1994; Geginat et al., 1999; Humann et al., 2007; Sashinami et al., 2010) and a recent study implicated specifically the N-terminal region in NK cell activation upon bacterial infection (Schmidt et al., 2011).

### Modification of cell envelope components

#### Peptidoglycan: acetylation and deacetylation

**Figure 3:**
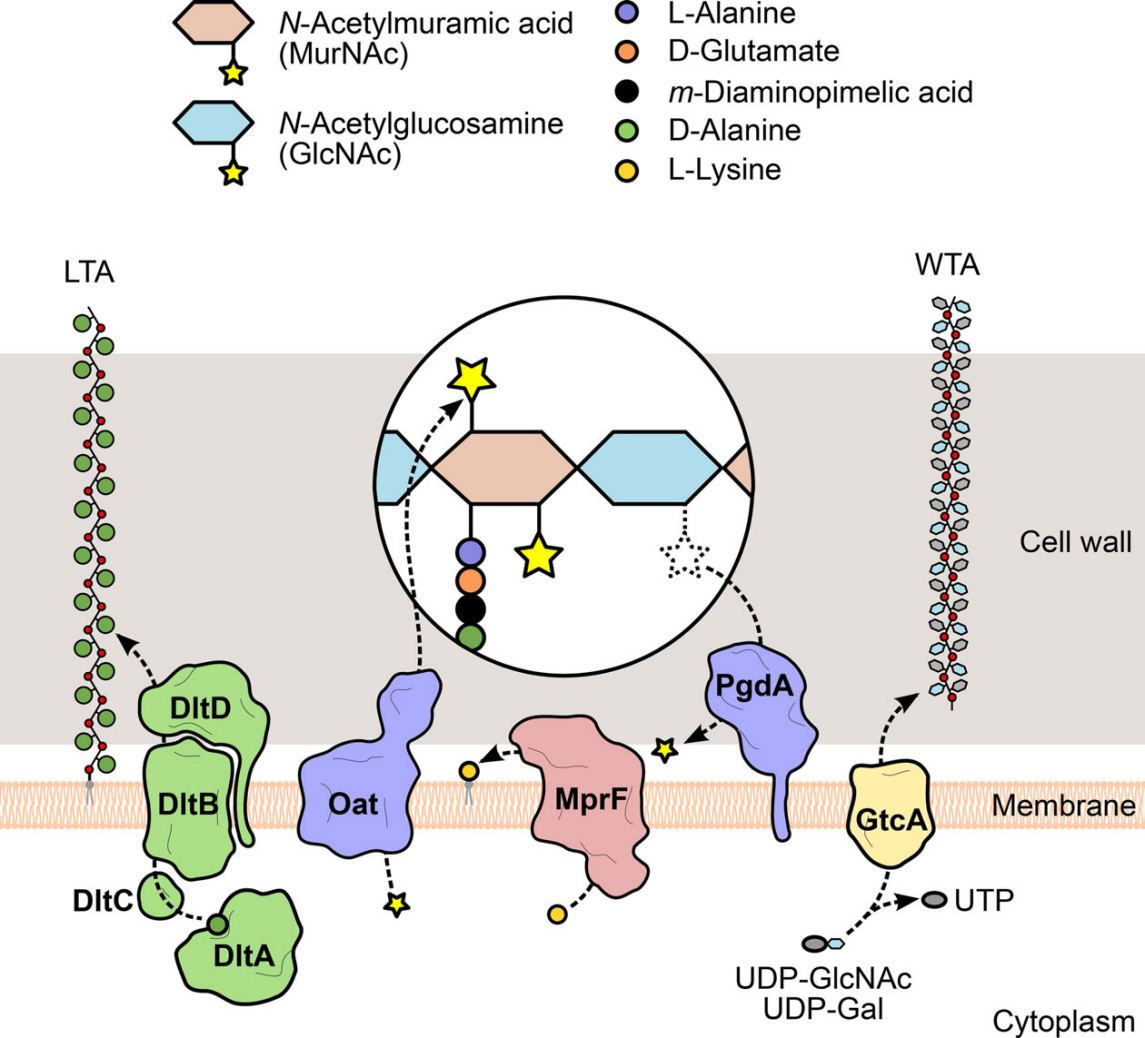
**Modification of *L. monocytogenes* cell envelope components.** To evade recognition and targeting by the host immune system, *L. monocytogenes* expresses surface proteins specialized in the introduction of fine modifications in cell envelope components. For instance, the addition of positively charged molecules contributes to the decrease of the overall negative charge of the bacterial surface, which in turn reduces the cell wall-binding affinity of cationic antimicrobial peptides. The D-alanylation of lipoteichoic acid (LTA) polymers by the DltABCD system (green) or the lysylation of plasma membrane phospholipids by MprF (pink) are examples of such approach. The glycosylation of wall teichoic acid (WTA) polymers was linked with listerial virulence (e.g., GtcA-mediated addition of GlcNAc in serotype 1/2a or galactose in serotype 4b), although the mechanism itself and the specific role of each sugar in this process are still unclear. Additionally, the peptidoglycan can be modified to become less recognizable or more resistant to degradation by host hydrolases (e.g., lysozyme) and prevent unwanted shedding of immunostimulatory muropeptides. These changes include the addition or removal of acetyl groups from the glycan chain amino sugars, catalyzed by two surface proteins, Oat and PgdA (purple), respectively.

Similar to autolysins, host-secreted hydrolases—such as lysozyme—bind to the bacterial cell wall and degrade the peptidoglycan. For this reason, they constitute one of the first and most important players of the host innate immune response against bacterial invaders. Because of their highly exposed peptidoglycan, Gram-positive bacteria are particularly susceptible, so they developed mechanisms to interfere with the activity of exogenous murolytic enzymes. In particular, the assembled peptidoglycan is modified by the addition of small molecules or large polymeric structures (Vollmer, 2008) (Figure 3). These changes prevent bacterial lysis and modulate the release of peptidoglycan fragments that can be recognized by specific host receptors and activate the innate immune response. For instance, the addition of *O*-linked (or removal of *N*-linked) acetyl groups to the peptidoglycan residues GlcNAc and MurNAc was shown to confer resistance to lysozyme and reduce the activation of the host immune response (Davis and Weiser, 2011). The deacetylation of GlcNAc and/or MurNAc is catalyzed by a deacetylase present in species containing *N*-deacetylated peptidoglycan (Vollmer, 2008). A significant proportion of the GlcNAc residues in the *L. monocytogenes* peptidoglycan was shown to be deacetylated, in a process exclusively dependent on the expression of PgdA (Boneca et al., 2007). In the absence of PgdA, *L. monocytogenes* is highly vulnerable to peptidoglycan hydrolases and cell wall-targeting antibiotics, dying rapidly inside macrophages and exhibiting attenuated virulence in the mouse model. Importantly, muropeptides derived from *N*-deacetylated peptidoglycan were less immunostimulatory than fully acetylated peptidoglycan fragments (Boneca et al., 2007; Popowska et al., 2009).

Compared to *N*-deacetylation, the mechanism of peptidoglycan *O*-acetylation has a more stringent specificity (only affects MurNAc residues), but it is a more predominant event, having been detected in numerous Gram-negative and Gram-positive species (Vollmer, 2008). This modification is enzymatically mediated by an integral membrane protein called *O*-acetyltransferase, which exports acetyl-containing substrates from the cytoplasm and transfers the acetyl group to the MurNAc residues in the assembled peptidoglycan strands (Clarke et al., 2002). First discovered in *S. aureus* (Bera et al., 2005), the gene coding for such an enzyme, *oatA*, was also identified in *L. monocytogenes* (Aubry et al., 2011). Analysis of mutants revealed that the activity of the listerial OatA largely overlapped with that of PgdA, as it is required for resistance to lysozyme and other antimicrobial compounds, survival within macrophages and virulence in mice (Aubry et al., 2011; Rae et al., 2011). However, while OatA-deficient strains induced the secretion of pro-inflammatory cytokines in mouse livers, particularly IL-6, PgdA mutants failed to stimulate IL-6 *in vivo* (Aubry et al., 2011), although they did so in macrophage cell lines (Boneca et al., 2007). The non-overlapping immunomodulatory activities of OatA and PgdA provide *L. monocytogenes* with a higher versatility in the control of the host immune response.

#### Secondary glycopolymers: LTA D-alanylation and WTA glycosylation

In addition to a myriad of surface proteins, the peptidoglycan of Gram-positive bacteria is densely decorated with a family of secondary glycopolymers called teichoic acids. These molecules generally consist of a polymeric backbone of phosphodiester-linked polyol repeats that is covalently bound either to the peptidoglycan matrix (wall teichoic acids, WTAs) or to the plasma membrane (lipoteichoic acids, LTAs), via a linkage unit connected to MurNAc residues or phospholipids, respectively. The abundance of phosphate groups confers strong anionic properties to teichoic acids, which increase the net negative charge of the bacterial surface (Neuhaus and Baddiley, 2003). The size of the polyol subunit and the presence and nature of substituent groups vary between and even within species, to the point of being used as serotype markers. While LTAs have conserved polyglycerol-phosphate backbones (Reichmann and Gründling, 2011), WTA monomers are chemically more diverse, the most common including glycerol- or ribitol-phosphate (Brown et al., 2013).

WTA/LTA subunits can be typically substituted with sugars or esterified with D-alanine, as a result of the action of specific glycosyltransferases or of the products of the *dltABCD* operon (Neuhaus and Baddiley, 2003). This operon encodes a multicomponent complex of cytosolic and membrane-bound proteins that transport D-alanine residues from the cytoplasm and incorporate them into extracellularly located teichoic acid polymers (Reichmann et al., 2013) (Figure 3). Given that D-alanine is positively charged at physiological pH, the addition of this molecule to teichoic acids represents a mechanism used by bacteria to fine-tune their surface charge in response to adverse environmental conditions. This process is particularly important for the protection against cationic antimicrobial peptides (CAMPs). Similarly to what was observed in *S. aureus* (Collins et al., 2002), failure to perform D-alanylation of *L. monocytogenes* WTAs due to genetic inactivation of the *dltABCD* operon results in a strain highly susceptible to several CAMPs and with significantly decreased virulence in the mouse model (Abachin et al., 2002). In addition, the mutant bacteria showed lower levels of adhesion, suggesting that the lack of D-alanylated WTAs, and likely the increased surface electronegativity, hinders bacterial attachment to host cells.

The similarities between WTAs and LTAs bring about a functional redundancy (Oku et al., 2009) that has complicated the understanding of the contribution of tailoring modifications to various aspects of bacterial physiology. The striking structural and biochemical diversity within WTAs, resulting from *O*-glycosylation of WTA monomers with a plethora of sugar molecules, provides additional complexity. Studies about the role of glycosidic substituents of WTAs have been mostly done in *S. aureus*, where the biogenesis and functions of teichoic acids have been better elucidated. They showed that sugar moieties confer immunogenic properties to WTAs (Juergens et al., 1963; Torii et al., 1964) and enable the binding of bacteriophages (Chatterjee et al., 1969). Interestingly, similar observations were made in *L. monocytogenes* (Wendlinger et al., 1996), which has only sugar-modified WTAs (Kamisango et al., 1983). This suggests an even more significant impact of WTA glycosylation in these processes. Evidences linking sugar modification of WTAs with *Listeria* virulence were obtained from studies using transposon-generated mutants. EGD (serotype 1/2a) mutants were screened in a mouse model for virulence attenuation (Autret et al., 2001) and multiple attenuated clones were found to contain an insertion in *gtcA*, a gene coding for a glycosyltransferase responsible for the tailoring of serotype 4b or 1/2a WTAs with galactose or GlcNAc, respectively (Promadej et al., 1999; Eugster et al., 2011) (Figure 3). In another study, the pathogenic potential of a serotype 4b *gtcA* mutant strain was shown to be strongly reduced in intragastrically infected mice. In addition, the absence of a functional GtcA protein decreased the ability to efficiently invade an enterocytic cell line, suggesting that GtcA-mediated WTA glycosylation in *L. monocytogenes* 4b is important for the intestinal phase of listeriosis (Faith et al., 2009).

#### Plasma membrane: phospholipid lysylation

The mechanisms of resistance to CAMPs by Gram-positive species can also include modifications of the plasma membrane, namely from the extracellular side, to mask the negative charge of the bacterial surface that favors the interaction with cationic peptides (Weidenmaier et al., 2003). Thus, in parallel with the D-alanylation of LTAs, the anionic surface environment can be reduced, for instance, by the covalent binding of positively charged L-lysine residues to the negatively charged head groups of phospholipids in the outer leaflet of the plasma membrane (Staubitz et al., 2004) (Figure 3). L-lysylated phospholipids were shown to occur in Gram-positive species (Nahaie et al., 1984; Fischer and Leopold, 1999) through a process dependent on the expression and enzymatic activity of the membrane protein MprF (multiple peptide resistance factor) (Peschel et al., 2001; Thedieck et al., 2006). This protein was first identified in *S. aureus*, where mutants lacking *mprF* showed growth and survival defects in the presence of CAMPs from diverse origins. This phenotype resulted from a stronger binding of CAMPs to the bacterial surface and was correlated with the absence of lysylphosphatidylglycerol (L-PG) from the membrane (Peschel et al., 2001), indicating that phospholipid L-lysylation promotes the repulsion of CAMPs. A functional ortholog of MprF was identified in *L. monocytogenes* and, like its staphylococcal version, is responsible for the generation of L-PG and for conferring resistance to CAMPs. Importantly, in the absence of MprF, entry levels in epithelial cell lines and *in vivo* virulence were significantly reduced (Thedieck et al., 2006), confirming the role of this surface modification protein in *Listeria* infection and resistance to host defense peptides.

## Transport systems

To survive and thrive in the host environment, *L. monocytogenes* developed specialized transport systems to enable the acquisition of essential nutrients, such as sugar, peptides or enzymatic cofactors, and to confer protection against aggressive stimuli from its surroundings, including osmotic shifts or toxic compounds. These systems consist of membrane-integrated substrate-binding and permease proteins that mediate the sensing, capture, and influx/efflux of specific substrates. A significant subset of these transport systems is actively expressed and assembled in the *L. monocytogenes* membrane during infection, and their presence at the surface was shown to be important for the survival and adaptation to the restrictive host conditions and, ultimately, for the overall process of pathogenesis. In this context, some of these systems are addressed in this section.

### Osmolyte uptake and bile acid extrusion

For most foodborne pathogens, the ability to sense and respond to the challenging environment of the gastrointestinal lumen is a key component of virulence. The osmolarity shift between the external environment and the small intestine triggers the synthesis of stress-related virulence factors, such as OpuC, an uptake system for carnitine, one of the most effective osmoprotectants in *L. monocytogenes* (Beumer et al., 1994). OpuC is essential for successful intestinal colonization and subsequent systemic infection (Sleator et al., 2001). In contrast, the two other osmolyte transporters involved in the uptake of glycine betaine, BetL and Gbu, appear dispensable for *Listeria* virulence (Sleator et al., 2000; Wemekamp-Kamphuis et al., 2002).

Following ingestion and gastric digestion, bile represents the most significant challenge for bacteria. *L. monocytogenes* was shown to be relatively resistant to bile (Begley et al., 2002), by inducing different bile resistance/detoxification systems, including a bile salt hydrolase (Bsh) important for colonization of the gastrointestinal tract (Dussurget et al., 2002). However, *L. monocytogenes* also expresses a bile exclusion system, BilE, which functions by actively extruding bile acids from the cell, inducing bile tolerance and the ability to infect mice via the oral route, a mechanism coordinately regulated by SigB (σ^*B*^) and PrfA (Sleator et al., 2005). Interestingly, osmolyte uptake systems appear to be required for the maintenance of *L. monocytogenes* bile tolerance, and the presence of carnitine seems to contribute to this process (Watson et al., 2009). In addition, genes involved in osmolyte uptake are responsive to bile salts, with *opuC* operon being highly expressed *in vitro* and *betL* in a mouse model of oral infection.

During replication in the cytosol of infected cells, *L. monocytogenes* uses two multidrug efflux pumps, MdrM and MdrT, to secrete the small second messenger cyclic-di-AMP (c-di-AMP) (Crimmins et al., 2008). Host recognition of c-di-AMP triggers the production of type I interferons, including IFN-β, which further promote *L. monocytogenes* virulence (Woodward et al., 2010). However, unregulated expression of MdrT was shown to significantly restrict virulence *in vivo*, by a yet unknown mechanism (Schwartz et al., 2012). Curiously, *L. monocytogenes* MdrM and MdrT are strongly induced by bile through a mechanism mediated by the BrtA transcriptional regulator, which has been previously shown to be important for *L. monocytogenes* virulence in mice (Crimmins et al., 2008). BrtA is a bile sensor that binds to the *mdrT* promoter de-repressing its transcription (Quillin et al., 2011). In addition to c-di-AMP, MdrT was also shown to work as an efflux pump for bile, acting in synergy with MdrM to induce bile resistance and promote colonization of the mouse liver and gallbladder.

### Nutrient uptake

*L. monocytogenes* is auxotrophic for selected vitamins and amino acids and thus must acquire them directly from the host (Welshimer, 1963; Premaratne et al., 1991; Marquis et al., 1993; Tsai and Hodgson, 2003). A total of 331 genes (11.6% of the genome) encoding transport proteins were identified in *L. monocytogenes* EGD-e (Glaser et al., 2001), reflecting the ability of *Listeria* to adapt to and colonize a broad range of ecosystems, including the human host. Approximately one third of these systems are devoted to the transport of carbon sources. Contrarily to non-pathogenic *Listeria* spp., *L. monocytogenes* is able to metabolize glucose-1-phosphate available in the host cell cytosol. This process is strictly dependent on PrfA (Ripio et al., 1997), which becomes fully activated when *Listeria* is in the cytoplasm (Freitag and Jacobs, 1999; Moors et al., 1999; Renzoni et al., 1999). *L. monocytogenes* was shown to exploit phosphorylated hexoses from the host cell cytosol as a source of carbon and energy for intracellular growth. Hexose phosphate uptake is mediated by the PrfA-regulated Hpt translocase, which is required for *Listeria* cytosolic proliferation and virulence in the mouse model (Chico-Calero et al., 2002).

In addition to using phosphorylated sugars, *L. monocytogenes* may use host cytosolic peptides as a source of amino acids during intracellular growth (Marquis et al., 1993). Three oligopeptide transport systems have been described as required for virulence in *L. monocytogenes*. OppA is an oligopeptide-binding protein encoded by the first gene of an oligopeptide permease (Opp) operon (*oppA*, *oppB*, *oppC*, *oppD*, and *oppF*) (Borezee et al., 2000). This ATP-dependent carrier is capable of transporting peptides with up to eight residues (Verheul et al., 1998). OppA was shown to mediate the transport of oligopeptides and to be involved in intracellular growth of *L. monocytogenes* in bone marrow-derived macrophages, but an *oppA* deletion mutant was only slightly less virulent than the wild type in the mouse model (Borezee et al., 2000). DtpT is a di- and tripeptide transporter shown to be required for growth when the essential amino acids leucine and valine were supplied as peptides. This transporter appears to be also involved in salt stress protection and to contribute to mouse model pathogenesis (Wouters et al., 2005). CtaP (for cysteine transport-associated protein) is the substrate-binding component of another oligopeptide transport system required for *L. monocytogenes* virulence (Port and Freitag, 2007). Other than cysteine transport, this multifunctional lipoprotein is associated with acid resistance, bacterial membrane integrity and adherence to host cells. In addition, a *ctaP* deletion mutant is severely attenuated following intragastric or intravenous inoculation of mice (Xayarath et al., 2009).

Thiamine pyrophosphate is an essential thiamine-derived enzymatic cofactor involved in central metabolism and amino acid biosynthesis. Because *L. monocytogenes* lacks the gene encoding ThiC, responsible for the synthesis of the thiamine precursor hydroxymethylpyrimidine, it is unable to synthesize thiamine in absence of this precursor (Schauer et al., 2009). However, the thiamine transporter ThiT was shown to be required for the uptake of this nutrient and, more broadly, for the intracellular growth of *L. monocytogenes*, indicating that thiamine acquisition is a critical step for bacteria proliferating in the host cell cytoplasm.

Successful pathogens obtain iron from the host environment. However, free iron is toxic at excessive concentrations and bacteria must regulate its accumulation (Stauff and Skaar, 2009). Whereas the host developed mechanisms for iron sequestration, pathogens engineered membrane transport systems for iron utilization during infection (McLaughlin et al., 2011). Iron acquisition is mediated by a number of systems that have been characterized in *L. monocytogenes*. In particular, it requires the activity of the putative ABC transporter encoded by the *hup* locus, since a mutant for *hupC* is impaired in heme uptake and shows decreased virulence (Jin et al., 2006). Interestingly, the SrtB-anchored protein SvpA seems to play a role in the capture of the iron porphyrin (Xiao et al., 2011). In most bacteria, including *L. monocytogenes*, iron homeostasis is controlled by the ferric uptake regulator Fur (Andrews et al., 2003). FrvA is a Fur-regulated virulence factor absolutely required for the growth of *L. monocytogenes* under iron-restricted conditions and for systemic infection. FrvA is required for the uptake of heme but is also essential for resistance to heme toxicity as well as maintenance of iron homeostasis. Sensitivity to heme toxicity may account for the significant attenuation of virulence during the systemic phase of infection in the murine infection model (McLaughlin et al., 2012).

## Regulation of cell surface-associated mechanisms

The spatial and temporal expression of bacterial cell envelope components, as well as the coordination between both processes, is crucial for their optimal function, in particular regarding virulence. These events are tightly controlled by a number of key regulators that sense specific stimuli from the host environment and/or the bacterium itself. The response resulting from the integration of all these signals allows *Listeria* to rapidly adapt its cell surface to the changing host conditions, maximizing its chances to survive and subvert the host cellular mechanisms for its own benefit.

### Transcriptional regulation

PrfA is the major *L. monocytogenes* transcriptional regulator of virulence determinants (Chakraborty et al., 1992; De las Heras et al., 2011). Mutants lacking a functional PrfA are unable to grow in infected cells and are almost avirulent in mice (Freitag et al., 1993). A recent study showed that PrfA activation is dispensable for vacuole escape but required for efficient bacterial dissemination and survival *in vivo* (Deshayes et al., 2012). The core PrfA regulon is composed of the ten virulence genes first identified as being PrfA-dependent (Scortti et al., 2007), seven of them being related with the bacterial surface (*actA, hly, inlAB, mpl*, and *plcAB*). In addition, nearly 160 other *L. monocytogenes* genes were shown to have their expression directly or indirectly dependent on PrfA. Among these genes, several encode virulence factors involved in *Listeria* cell envelope architecture, composition, and modification (*dtpT, frvA, hpt, inlH, lapB, opuC*, and *prsA2*) (Ripio et al., 1998; Dussurget et al., 2002; Milohanic et al., 2003; Raynaud and Charbit, 2005; Marr et al., 2006; Reis et al., 2010). PrfA activates transcription by binding to a PrfA box, a palindromic sequence (tTAACanntGTtAa) in the promoter of the target gene (Scortti et al., 2007; Freitag et al., 2009). PrfA integrates both environment- and bacteria-elicited signals to ensure the proper spatiotemporal transcription of its regulon. The expression of PrfA itself is simultaneously controlled by an RNA thermosensor mechanism that enables translation of the *prfA* mRNA only at temperatures close to 37°C, and by a trans-acting riboswitch (Johansson et al., 2002; Loh et al., 2009). An unstructured 5'-coding region of the *prfA* mRNA was recently identified as required for efficient translation (Loh et al., 2012). Its activity is postulated to be regulated through an allosteric shift mediated by a cofactor yet to be identified. Notwithstanding, the positive charge within the PrfA binding pocket was shown to contribute to the intracellular activation of PrfA, presumably by facilitating the binding of an anionic cofactor (Xayarath et al., 2011). Links between carbon metabolism and PrfA-dependent transcription suggest that host nutrient availability may work as an intracellular localization signal for *L. monocytogenes*, ensuring the strongest induction levels in the host cell cytoplasm and repression outside of the host environment (Freitag et al., 2009; Eisenreich et al., 2010).

σ^*B*^ is the major regulator of the class II stress response genes. Several transcriptomic and proteomic analyses revealed that σ^*B*^ regulates a large and diverse set of genes (nearly 200) predicted to function in stress tolerance, carbohydrate metabolism, transport, and also in cell envelope processes and virulence (Hain et al., 2008; Mujahid et al., 2013). In particular, a great number of genes related to bacterial surface architecture and maintenance, and involved in virulence, are regulated by σ^*B*^ (*actA, bilE, chiA, dtpT, hly, iap, inlABH, lapB, lpeA, opuC, plcAB, prfA, prsA2, sigB*). Interestingly, a significant subset of these genes is co-regulated by PrfA (*actA, dtpT, hly, inlABH, lapB, opuC, plcAB, prfA, prsA2)*. PrfA and σ^*B*^ were shown to jointly contribute to processes such as intracellular growth and virulence (Nadon et al., 2002; Kazmierczak et al., 2006; Chaturongakul et al., 2011). In addition, evidences suggest that σ^*B*^ fine-tunes *prfA* expression inside host cells to avoid overexpression of virulence genes that may compromise the host cell (Ollinger et al., 2009).

VirR is the response regulator element of the VirR/VirS two-component system. VirR was shown to be required for efficient mouse liver colonization and to positively control the transcription of 17 genes (Mandin et al., 2005), among them, the surface-related *dlt* operon and *mprF* (see Modification of Cell Envelope Components). The fact that VirR regulates the expression of both *dltABCD* and *mprF* genes suggests that the VirR/VirS system plays a role in the modulation of *L. monocytogenes* resistance against host cationic peptides and constitutes another important virulence regulon involved in *Listeria* surface adaptation and pathogenesis.

Flagellar motility is an essential mechanism by which bacteria can adapt to and survive in diverse environmental niches. Although flagella confer an advantage to *L. monocytogenes* for host colonization (Dons et al., 2004; Bigot et al., 2005; O'Neil and Marquis, 2006), listerial flagellin also stimulates host innate immune responses (Hayashi et al., 2001). Consequently, at the physiological temperature of the host (37°C), *L. monocytogenes* shuts down flagellar motility, repressing expression and assembly of flagellar components. Also required for virulence, this down-regulation is governed by the reciprocal activities of the MogR transcriptional repressor and the bifunctional flagellar anti-repressor/glycosyltransferase GmaR, which is kept activated by the orphan response regulator DegU, at temperatures under 30°C (Gründling et al., 2004; Shen and Higgins, 2006; Kamp and Higgins, 2009). Recently, GmaR was shown to function as a thermosensitive anti-repressor that integrates temperature signals into transcriptional control of flagellar motility (Kamp and Higgins, 2011).

### Spatiotemporal regulation of surface proteins

#### In vivo regulation

**Figure 4:**
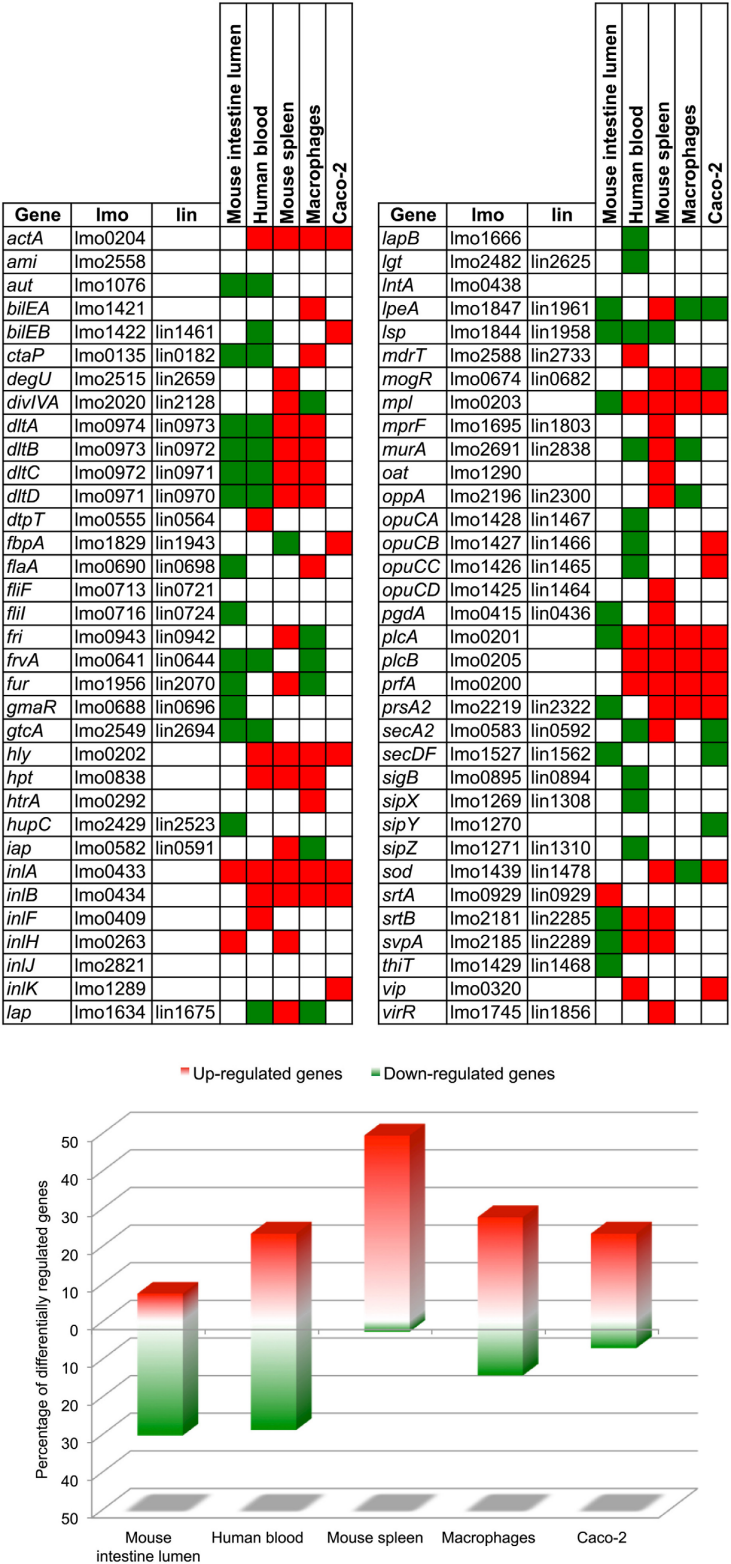
***In vivo* and intracellular expression of virulence genes involved in *L. monocytogenes* surface architecture and modification.** In each condition, genes are shown up-regulated (red) or down-regulated (green) relative to the corresponding transcription levels during exponential growth in standard culture conditions (BHI broth, 37°C). Data were compiled from different transcriptomic studies that used the same model strain and reference condition (Chatterjee et al., 2006; Joseph et al., 2006; Camejo et al., 2009; Toledo-Arana et al., 2009). lmo, gene number in *L. monocytogenes* EGD-e; lin, ortholog gene number in *L. innocua* CLIP 11262.

Virulence is by definition expressed in a susceptible host and involves a dynamic crosstalk with the pathogen. In response to the host environment, *L. monocytogenes* tightly regulates and coordinates the expression of virulence factors to promote an efficient infection (Chatterjee et al., 2006; Joseph et al., 2006; Camejo et al., 2009; Toledo-Arana et al., 2009). This is particularly the case for virulence factors involved in the modification of the bacterial surface (Figure 4). In the mouse intestinal lumen, most of these genes are down-regulated as compared to exponential growth *in vitro*, suggesting that they are either needed only for later stages of infection or their premature expression hinders the progress of infection in the gastrointestinal phase. Reversely, when infecting the mouse spleen, *L. monocytogenes* overexpresses most of the virulence genes related with bacterial surface architecture and modification. Globally, there is a good correlation between the expression status of these genes in the different *in vivo* conditions analyzed and the role of the encoded proteins. For example, the only surface factors up-regulated by *L. monocytogenes* in the mouse intestinal lumen are InlA, InlH and SrtA, possibly to prepare bacteria for the invasion of enterocytes. When inside host cells, *L. monocytogenes* expresses genes required for vacuole escape, intracellular motility and multiplication (Figure 4). This reveals the ability to fine-tune the expression of the factors involved in *Listeria* surface architecture and modification in response to rapidly changing environmental conditions, particularly in accordance with the infection phase. Interestingly, the characterization of the cell wall proteome of bacteria proliferating within eukaryotic cells revealed that that the adaptation of *L. monocytogenes* to the intracellular lifestyle involves changes in the relative abundance of certain surface proteins, such as InlA and InlH (García-Del Portillo and Pucciarelli, 2012).

#### Spatial regulation

Besides the transcriptional control of virulence-associated factors, the regulation of their localization is also essential to ensure a successful infection. As already mentioned here, *L. monocytogenes* uses different mechanisms to target to its surface and secrete proteins that contribute to the colonization of host tissues.

Many listerial virulence effectors (InlA, InlF, InlH, InlJ, InlK, Vip, and SvpA) are covalently attached to the peptidoglycan (see Sortases). Recently, the three-dimensional localization of InlA, InlH, InlJ, and SvpA in the cell envelope of *Listeria* grown at different conditions was reported (Bruck et al., 2011). In this study, peptidoglycan-anchored proteins were found positioned along the lateral cell wall in non-overlapping helices, but could also be localized at the poles and distributed asymmetrically when specific regulatory pathways were activated. For instance, whereas InlA and InlJ (SrtA substrates) are enriched at the bacterial poles during exponential growth, InlA and InlH relocate to the septum when entering stationary phase or under oxidative stress. It was proposed that, in response to PrfA activation, excess of InlA protein may saturate sidewall anchor points and thus reach the septum (Bierne and Dramsi, 2012). The fact that *inlAH* are also under the control of σ^*B*^ could suggest an interconnection between cell wall protein anchoring and σ^*B*^-dependent stress response. This is reinforced by a recent study showing that the activation of InlA and InlH during the transit of *L. monocytogenes* toward stationary growth phase is dependent on the functionality of SrtA (Mariscotti et al., 2012). In contrast, SvpA (SrtB substrate) is present at the poles and excluded from the septum under conditions of low iron concentration. This suggests that *L. monocytogenes* can reorganize the spatial localization of its surface virulence factors in response to environmental changes, to best accommodate to the particular conditions of the each stage of the infectious process. SrtA links LPXTG proteins to the peptidoglycan precursor lipid II, whereas SrtB is proposed to anchor substrates directly to the mature peptidoglycan (Marraffini and Schneewind, 2005), suggesting that *Listeria* uses different sortases to anchor proteins to distinct sites of the bacterial surface.

The polar distribution of ActA on the *L. monocytogenes* surface is required for actin-based motility and successful infection (see Hydrophobic Tail) and appears to be a consequence of different cell wall growth rates along the bacterium, of the relative rates of protein secretion and degradation, and of bacterial growth (Rafelski and Theriot, 2006). Interestingly, although anchored to the membrane, ActA was shown to be specifically co-purified with the peptidoglycan fraction isolated from intracellular bacteria (García-del Portillo et al., 2011).

DivIVA is a crucial topological factor required for completion of cell division in *L. monocytogenes*. It not only affects *Listeria* cell separation, but also biofilm formation, host cell invasion and virulence. DivIVA was shown to influence the activity of the Sec system alternative ATPase SecA2, resulting in reduced extracellular levels of the autolysins p60 and MurA and inducing a pronounced chaining phenotype (Halbedel et al., 2012).

All these observations clearly demonstrate how *Listeria* developed a complex regulatory network that links cell growth, cell wall dynamics, cell wall protein anchoring and response to environmental conditions, and coordinates the spatiotemporal expression and activity of surface-associated virulence factors.

### Conflict of interest statement

The authors declare that the research was conducted in the absence of any commercial or financial relationships that could be construed as a potential conflict of interest.
